# Long-term oriented culture, performance pressure and corporate innovation: Evidence from China

**DOI:** 10.1371/journal.pone.0302148

**Published:** 2024-05-21

**Authors:** Qiaoling Fang, Cai Wen, Hui Xu

**Affiliations:** 1 School of Economics and Management, Fuzhou University, Fuzhou, Fujian, China; 2 School of Management, Zhaotong University, Zhaotong, Yunnan, China; 3 School of Accounting, Yunnan University of Finance and Economics, Kunming, Yunnan, China; Yunnan Technology and Business University, CHINA

## Abstract

This paper extracts culture element of long-term orientation from Chinese listed firm’s annual report, then argue and testify whether long-term orientation can help firms to hang on risky decision especially as innovation, when firms are under performance pressure. There are three main conclusions. First, we report that the higher degree of long-term oriented culture a firm has, the stronger innovation capability the firm shows. Second, we find that long-term oriented culture can improve employee’s educational qualification to promote corporate innovation, as well as improve the corporate internal control to promote innovation. Third, when firms are subjected to internal or external performance pressure in their business process, higher long-term oriented culture will make firms more innovative.

## 1.Introduction

Culture has economic consequence [1]. With the development of culture and finance, the study of culture on corporate decision making has received much attention in the last decade [2], including culture affecting financial reporting practices [3,4], accounting conservatism [5], earnings discretion [6], corporate risk taking [7], and different kinds of corporate investments [8,9]. Studies have usually measured culture by cultural elements [8,10], which at the very beginning, contained four dimensions, namely individualism, uncertainty avoidance, power distance, and masculinity. To account for the cultural characteristics of Eastern countries, Hofstede [10] supplemented these with a fifth culture dimension, that of long-term orientation. Scholars believe that the long-term orientation was one of the main reasons for the boosting in the economies of East Asian countries in the late 20th century.

Long-term orientation refers to acceptance by members of a given culture of delays in meeting their material, emotional, and social needs, or whether these people tend to judge their behavior in a future-oriented manner. Although the typical cultural characteristics of Eastern cultures cannot be ignored [10], there are very few related studies that only concentrate on how long-term oriented culture affects family firms [11,12]. One of the probable reasons is that the country-specific cultural elements can only be applied in multinational studies; therefore, it is hard to measure long-term oriented culture when only considering one specific country’s firms. Some Eastern cultures, such as Confucian, exhibit long-term orientation as well, and Confucianism also has the characteristic of high uncertainty avoidance. This makes it hard to evaluate whether corporate risk decision is affected by Confucianism due to long-term orientation or uncertainty avoidance. In accordance with Li et al. [13], we extracted the cultural element of long-term orientation from the annual reports of Chinese listed firms, then argued and testified whether long-term orientation can help firms to hang onto risky decisions, especially on innovation, when firms are under performance pressure.

We conducted our investigation in China for three reasons. First, China is a country that is deeply affected by informal regulation. In particular, China ranked first in long-term orientation on the country culture index survey [10]. Affected by long-term orientation, people will focus more on long-term goals than on the achievement of immediate benefits. China’s recent social and economic development concepts, such as sustainable development, "carbon neutrality" and "the gold and silver mountains are not better than the green mountain" are all practical manifestations of long-term oriented culture. Second, Chinese listed firms are under greater performance pressure. Based on Chinese stock market and management regulations, a firm that has two consecutive years of negative profits is subject to mandatory delisting. Listed firms need to keep positive sales and profit to retain the status of listed, which means a firm has a strong motive to give up huge expenses or investments, including innovation expense. Third, Chinese innovation inputs and outputs have maintained rapid growth in the last decade [14], and Chinese total investment in R&D has increased to 2.4 trillion. In the next five years, the Chinese government plans to maintain an average annual growth rate of "more than 7%" in the scientific and technological resources of the whole society. Under the conditions of strong Chinese policy incentives for innovation, and performance pressure, we investigated how long-term oriented culture affects innovation.

We obtained several findings. First, we distinguish innovation capabilities into substantive innovation, such as patents for invention, and strategic innovation, such as patents for non-invention. Measuring long-term culture from the text of a firm’s annual report, we find that for both substantive and strategic innovation, the higher the degree of long-term oriented culture a firm has, the stronger innovation capability the firm shows. This conclusion remains robust through a serious of robustness tests, including replacing the measurement of long-term culture, the use of lagged culture variables, and the use of instrument variable and PSM to settle endogeneity problems.

Second, we obtained findings related to specific paths. We show that firms with higher long-term oriented culture usually pay more attention to cultivating employee skills and development, while employee educational qualifications are significantly and positively related to innovation capability. Thus, a long-term oriented culture can improve employee educational qualifications to promote corporate innovation, especially substantive innovative capacity rather than strategic innovative capacity. In addition, firms with higher long-term oriented culture focus more on sustainable development, which requires a good internal control system for support. Therefore, long-term oriented culture can improve the internal control system to promote both kinds of innovation.

Third, an extended analysis shows that when firms are subjected to different kinds of performance pressure in their business processes, including inner performance pressure such as low profit and external performance pressure such as fierce market competition or high analyst tracking, firms with a higher long-term oriented culture will be more innovative in both kinds of innovations.

We make the following contributions to the literature. First, we expand the literature on Culture and Finance. Although related research has widely discussed how different kinds of culture affect risky corporate decisions [7,8,15,16], little attention has been placed on how Eastern culture, especially long-term orientation, affects firm decisions. A possible reason is that long-term orientation is hard to accurately measure. According to the measurement of other cultural elements [13], we extracted cultural element of long-term orientation from the annual reports of Chinese listed firms to document that long-term culture promote innovation. Our paper complements the literature on the economic consequences of long-term culture [11,12] by including all listed firms and from the viewpoint of corporate innovation.

Second, we advance the literature on corporate innovation. Innovation is affected by both institutional [17] and informal [18] systems. On the one hand, according to institutional background of China, firms are more likely to be under performance pressure and to reduce innovation. On the other hand, China is generally affected by long-term oriented culture, Chinese firms tend to insist on innovation. Since informal system, such as culture, plays an important role in shaping accounting systems [3,4,19], while also influencing financial decisions of enterprises. It may be a substitute mechanism when there exists weak formal systems in the capital market, we choose China, which is the largest emerging market to test our story. Our findings not only contribute to the existing literature [18,20] on how culture affect innovation from the view of long-term orientation, but also expand the innovation literature by proposing that interactive effect of both institutional and culture factors can affect corporate innovation.

Last, long-term oriented culture not only exists in China but also in other emerging eastern countries. It has generally been seen as one of the main reasons for the boost in the economies of East Asian countries in the late 20th century. How long-term oriented culture affects corporate decisions deserves to be thoroughly investigated. Hence, this paper offers valuable lessons on the economic consequences of culture in other emerging market countries.

## 2. Literature and hypothesis

### 2.1 Culture and corporate innovation

In the fields of finance and accounting, informal rules, such as culture, plays an important role in shaping accounting systems [3,4,19] and making corporate financial decisions for enterprises [2]. Culture can change the behavioral patterns of rational economic agents, which in turn leads to differences in economic consequences [1], such as corporate innovation.

Related research about national culture shows that it plays an important role in influencing global corporate innovation [18]. Countries with high individualism show overall characteristics of overconfidence [21] and high innovation-related social rewards [22], thus, they tend to promote innovation [18,20]. Countries with high levels of uncertainty avoidance are less tolerant of failure and inhibit innovation [18,23]. There is inconsistent evidence on power distance, which may promote [24] or inhibit [25] innovation.

### 2.2 Long-term oriented culture and corporate innovation

Long-term orientation is one of the multidimensional aspects of culture [10]. In long-term oriented countries, people focus more on long-term goals than on the achievement of immediate benefits, and they are willing to devote themselves to long-term goals. Therefore, the economic consequences of a long-term oriented culture are generally manifested by a controlling shareholder’s goal of maximising the long-term value of the firm [26] and the management’s avoidance of short-sighted behaviors and decisions. Chinese culture is typically long-term oriented. According to the country culture index survey [10], China ranks first in the world in the long-term orientation dimension. Lumpkin and Brigham [27] argued that the positive effects of a long-term oriented culture on firms can be divided into three dimensions: futurity, sustainability, and resilience. From these three dimensions, we argue that firms with a long-term oriented culture are more innovative for the following reasons:

First, firms with a high long-term oriented culture pay more attention to future development. Thus, firms are more inclined to make decisions that may not necessarily be beneficial to performance in the short term but will contribute to the achievement of long-term corporate goals and sustainability. Innovation is a very typical long-term decision. When a company makes a risky decision to adhere to innovation, it needs to invest a lot of resources in the short term, including capital or manpower, but hardly expects to get an immediate return. After years of sustained and huge investment in R&D expense, the output of innovation not only improve firm’s long-term performance but also increase technological and competitive advantages in the long run, finally helping the firm to achieve sustainable development.

Second, a long-term oriented culture will enhance a firm’s failure tolerance. Innovation activities are characterized by multiple periods, uncertainty, and high failure rates, requiring a firm to better tolerate failure [28]. A firm with a long-term oriented culture does not expect immediate output, creativity requires sufficient time for reflection and cultivation, and a longer and continuous time horizon may help firms to sustain exploratory research. A long-term oriented culture is not only more tolerant of failure in risky activities [29], which reduces the pressure on management and technicians, but also continuously invests resources over a long period of time, which helps companies to initiate and sustain their innovation.

Third, a long-term oriented culture is characterized by resilience, perseverance and responsibility. Firms can decide whether to maintain or update their existing productive operations. Updating requires more to be invested in innovation. Future development and failure tolerance also requires firms to be resilient and to persevere with innovation before they get innovative performance. Accordingly, hypothesis 1 is formulated:

*H1*: *The higher the degree of long-term oriented culture a firm has*, *the higher the innovation capacity the firm shows*.

### 2.3 Long-term oriented culture and corporate innovation: Paths analysis

Based on H1, the more important question is: What are the specific paths by which long-term oriented culture to improve innovation? We consider the following two aspects:

*The path of human resources deveploment*. A firm with a long-term oriented culture is likely to place more value on skills and knowledge [30], therefore, it will pay more attention to development talent teams and is more likely to hire well-educated employees. It has been documented that employees’ education has a positive impact on corporate innovation. For example, Bantel and Jackson [31] found that when an executive team has a higher education level, the bank will have better innovation. Jiang et al. [32] found that teamwork significantly and positively influences team innovation by virtue of learning from mistakes based on the human resources of all employees, with team human resource encompassing all education, skills and abilities of a team member. Therefore, we believe that firms with a long-term oriented culture can enhance their innovation capabilities by strengthening human resources. The quality of human resources is one of the paths by which long-term oriented culture influences innovation ability.

*The path of internal control*. Long-term oriented culture makes firm pay more attention to the future, sustainability and resilience. A good internal control system can enhance the sustainability of a firm [33], and firms with a long-term oriented culture are more inclined to establish a solid internal control system. The innovation process faces higher uncertainties along with a higher risk of failure [34]. Although a long-term oriented culture gives a firm a higher tolerance for failure, a weak internal control system, such as insufficient risk assessment and inadequate control and preventive measures, will be bad for a firm’s performance and even its long-run development and, thus, will impede innovation. A solid internal control system can regulate and supervise corporate investment behavior, smooth internal information flow, reduce information asymmetry among stakeholders, and then effectively control innovative risk and improve innovative output. Besides, agency problems between managers and shareholders will lead to investment inefficiency, thus impeding innovation. A good internal control system enables the monitoring of executive behavior and inhibits adverse selection, thereby contributing to innovative activities that promote the strategic development of the firm. Therefore, we believe that the quality of internal control is also one of the paths by which long-term oriented culture influences innovation ability. Accordingly, hypothesis 2 is proposed:

*H2a*: *By improving or strengthening human capital*, *a higher degree of long-term oriented culture enhances firm innovation*.
*H2b*: *By improving or strengthening internal control*, *a higher degree of long-term oriented culture enhances firm innovation*.

### 2.4 Long-term oriented culture, performance pressure and corporate innovation

We also consider the performance pressure. Existing research proved that short-term financial performance pressure is a major obstacle to innovation [33]. There is a special factor that creates pressure on firms in China, namely that the Chinese stock market and management regulations mandate that two consecutive years of negative profits will result in delisting. Listed firms need to keep good revenue and positive profit to retain the status of listed, which means Chinese listed firms usually face more performance pressure.

From the view of inner performance pressure, listed firms need to keep positive performance year by year, which means a firm has a strong motive to give up huge expenses or investments, such as innovation expense, to avoid negative financial performance. However, long-term oriented culture makes firms more tolerant of failure [29]. The high risk of innovation usually results in a higher risk of failure as well as poor short-term performance.

From the view of external performance pressure, innovation also brings higher information asymmetry due to unknown outcomes, making it difficult for investors to accurately assess firm value [35]. Performance decline or innovation failure will attract external stakeholder attention, which, in turn, creates external pressure. For example, pressure from fierce market competition or high analyst tracking may force managers to choose safer, more secure projects that are not conducive to innovation [36]. But long-term oriented culture is more likely to play its governance role as informal rules. Firms influenced by higher long-term oriented culture will be more tolerant of the failure of firm members in risk-taking activities, reducing the pressure on management and R&D teams, which in turn reduces the tendency of managers to cut investment in innovation. Accordingly, research hypothesis 3 is stated:

*H3*: *When under higher performance pressure*, *firms with a higher long-term oriented culture are more innovative*.

## 3. Sample, research design, and descriptive statistics

### 3.1. Data sources and sample selection

We selected A-share listed firms from 2008 to 2019 as the initial research sample. We then deleted financial firms, special treatment firms and firms with an asset-liability ratio greater than 1. The final sample had 15,324 firm-years. The corporate long-term oriented culture indicators were obtained from annual reports, which were obtained by manual reading and PYTHON text analysis method; patent data were obtained from the Chinese Research Data Services (CNRDS) database; and other financial and governance data were obtained from the China Securities Market and Accounting Research (CSMAR) database.

### 3.2. Variable definition

#### 3.2.1 Long-term oriented culture

Previous literature has used the Hofstede or Schwartz Country Culture Index [7] or the number of religious temples within a certain distance [37] to measure corporate culture. However, the Country Culture Index [7] is difficult to apply to research within a certain country, Chinese citizens do not have a universal understanding of religious consciousness, and there is a significant subjective bias in cultural scoring methods. Therefore, we considered it more reasonable to measure corporate culture by extracting vocabulary from annual reports through text analysis. Annual reports have rich textual information that better reflects the cultural characteristics of the firms. The continuous, annual textual sources can timely reflect changes in the culture of the firms. Therefore, we used text analysis to extract the vocabulary of culture in the annual reports. The steps that were taken to establish the main indicators of firms’ long-term oriented culture are as follows:

First, we built a dictionary. According to Li et al. [13], in order to measure the degree of a firm’s long-term oriented culture, we first needed to construct a dictionary of long-term oriented culture. To this end, we referred to the long-term oriented cultural words mentioned in Hofstede et al. [30] and Brighamet et al. [11] to construct an English dictionary, further translate the English dictionary into a Chinese dictionary, and search for similar words based on the Modern Chinese Dictionary, thereby establishing the basic framework of a long-term oriented cultural dictionary. Considering the complexity of Chinese sentences, we did not use machine learning method, but instead used manual reading to go through the annual reports and determine the final dictionary. Specifically, we based our examination on the text of the management discussion and analysis (MD&A) section of annual reports disclosed by listed firms; next, we organized two groups of members, and each group selected one hundred text segments from the annual reports of listed firms, manually read them based on the basic framework of the long-term oriented cultural dictionary, then collected similar words; finally, we summarized the vocabulary obtained from the two groups and established a long-term oriented cultural dictionary for the firms, as shown in Table 1.

**Table 1:** The words of long-term oriented culture.

Chinese words of long-term oriented culture	Corresponding English translation words
一直、一如既往、均衡、惠民生、良性发展、综合、巩固、恒久、不断完善、延续、核心竞争力、工匠精神、深化改革、深远、深入、做精做细、环境效益、长青、进一步深化、循序渐进、绿色、和谐一致、健康发展、百年、提质、持久、精益生产、长久、长效、长远、长治久安、长期、保持、有序推进、整体竞争、整体提升、永续、规划、公益事业、保质、品质、质量发展、有质量的增长、质量增长、理性、科学发展、平稳有序、扎实、坚实基础、稳定、稳固、稳步、稳健、平稳增长、稳中求进、稳中有进、稳中有升、稳中有为、稳中向好、加强基础管理、强基础、持续、大局	always, balanced, benefit the people’s livelihood, benign development, comprehensive, consolidation, constant, constant improvement, continuity, core competitiveness, craftsmanship, deepen reform, far-reaching, deeper, doing fine and fine, environmental benefits, evergreen, further deepening, gradual and orderly, green, harmony, healthy development, hundred years, improve quality, lasting, lean production, long term, long term, long term stability, maintain, orderly advancement, overall competition, overall improvement, perpetually, planning, public welfare, quality, quality development, rationality, scientific development, smooth and orderly, solid, solid foundation, stable, steady, steady growth, steady progress, steady to good, strategic, strengthening basic management, strong foundation, sustainability, the big picture

Second, we engaged in text analysis. Using Python software and the "Jieba" segmentation algorithm, manually selected words were added to the vocabulary of the "Jieba" algorithm. The corresponding text segments in the annual report of listed firms were segmented, and the number of long-term oriented cultural words that appeared in the company year dimension was calculated.

Third, we designed the variables. Based on the number of long-term oriented cultural words obtained from the previous steps, we constructed the main variable (*Culture_LT*) of long-term oriented culture at the firm-year level, which is the number of long-term oriented cultural words add 1, and then take the natural logarithm. In addition, we needed to ensure the robustness of the empirical results due to the possibility that the textual information in the annual report of firms may be influenced by the year, industry, and estimation errors, and thus, we designed the variable *Culture_LT_5*, which divides *Culture_LT* into 1, 2, 3, 4 and 5 levels according to the long-term oriented culture level of the industry in which the firm is located every year.

#### 3.2.2. Corporate innovation

We measure the innovation capacity of firms as the total number of at domestic and foreign patent applications, then add 1, and then take the natural logarithm following Dosi et al. [38], Hall and Harhoff [39] and others. The reasons for using patent applications to measure the innovation of firms are that: on the one hand, the number of patent applications is a more reasonable proxy for the innovation capability of a company since patent applications are the final output of the resources invested in technological innovation; in addition, compared with the number of patents granted, the number of patent applications is a more reasonable measure of corporate innovation. Patent technology is often applied to production or operations during the application process, which, in turn, affects corporate performance. However, after the patent is granted, it needs to be tested according to the regulations and an annual fee must be paid. At the same time, the patent is also vulnerable to factors such as government rent seeking. Therefore, the number of patent applications is more stable, reliable and timely than the number of patents granted. The reasons we did not use R&D expenditure as a measure of corporate innovation is that R&D expenditure can only represent the level of R&D input, but it can hardly represent R&D output, which means innovation. Thus, in these years, a China study usually used patent-related indexes to measure innovation [40].

Li and Zheng [41] distinguished innovation into substantive and strategic innovation behaviors from the perspective of innovation effectiveness. Substantive innovation belongs to high-tech innovation and can promote social and technological development, while strategic innovation is a relatively low level of innovation and is only aimed at catering to government policies. In view of this, we also distinguished corporate innovation into substantive and strategic innovation. Substantive innovation is the total number of invention patent applications, then add 1, and then take the natural logarithm. Strategic innovation is the total number of non-invention patent applications including utility models and design patent applications, then add 1, and then take the natural logarithm. The Table 2 presents the definitions of the variables.

**Table 2:** Variable definitions.

Variable	Definition
***Main regression model variables***	
*Patent*	The total number of domestic and foreign patents applied for, and add 1, and then take the natural logarithm.
*Patent_Sub*	The number of domestic and foreign invention patents applied for, and add 1, and then take the natural logarithm.
*Patent_Str*	The number of domestic and foreign utility model patents and design patents applied for, and add 1, and then take the natural logarithm.
*Culture_LT*	The the total words of the long-term oriented culture of firm’s annual report, and add 1, and then take the natural logarithm.
*Culture_LT_5*	*Culture_LT_5* divides *Culture_LT* into 1, 2, 3, 4 and 5 levels according to the long-term oriented culture level of the industry in which the firm is located every year.
*DumCulture_LT*	An indicator variable equal to 1 if the *Culture_LT* is larger than the average value in the industry each year where the firm located, otherwise is 0.
***Intermediate variables***	
*MasterRatio*	Percentage of firm’s employees with a master’s degree.
*IC*	Internal Control Index published by Diebold divided by 100.
***Performance pressure variables***	
*P_Roa*	An indicator variable equal to 1 if the firm’s ROA is less than the average value in the industry each year where the firm located, otherwise is 0.
*P_HHI*	The Herfindahl index is used to measure product market competition. *P_HHI* is an indicator variable equal to 1 if the firm’s Herfindahl index is less than the average value in the industry each year where the firm located, which means the firm’ product market competition is is more intense, otherwise is 0.
*P_Analyst*	The total number of analysts tracked, and add 1, and then take the natural logarithm.
***Control variables***	
*Lage*	The natural logarithm of the years from the company listed
*Roa*	The value that net income divided by total assets.
*Top10*	The percentage of shares held by the top ten shareholders.
*Ocf*	The net cash flow from operating activities divided by total assets
*Growth*	The ratio of the sales revenue growth.
*Indep*	The number of independent directors on the board of directors divided by the total number of directors
*Lev*	The ratio of liability to total assets.
*Size*	The natural logarithm of total assets.
*Board*	The natural logarithm of the total number of board members.
*Dual*	An indicator variable equal to 1 if the chairman and CEO is one person, otherwise it is 0
*Soe*	An indicator variable equal to 1 if the firm control by the state, otherwise is 0.
*RD*	The ratio of the R&D expenditure to total assets.

### 3.3. Research design

#### 3.3.1. Hypothesis 1: The long-term oriented culture and corporate innovation

Referring to Cornaggia et al. [42], the following basic model was designed to test the effect of long-term oriented culture on corporate innovation:

$${Innovation}_{i,t+1}=\alpha_{0}+\alpha_{1}{Culture\_LT}_{i,t}+\alpha_{2}{Lage}_{i,t}+\alpha_{3}{Roa}_{i,t}+\alpha_{4}{Top10}_{i,t}+\alpha_{5}{Ocf}_{i,t}+\alpha_{6}{Growth}_{i,t}+\alpha_{7}{Indep}_{i,t}+\alpha_{8}{Lev}_{i,t}+\alpha_{9}{Size}_{i,t}+\alpha_{10}{Board}_{i,t}+\alpha_{11}{Dual}_{i,t}+\alpha_{12}{Soe}_{i,t}+\alpha_{13}\sum {Year}_{i}+\alpha_{14}\sum {Ind}_{i}+\varepsilon_{i,t}$$ (1)

where *Innovation* is main dependent variable and is represented by three variables: Patent, *Patent_Sub* and *Patent_Str*. Among them, *Patent* is the total number of patent applications at home and abroad, then add 1, and then take the natural logarithm. To order to test the specific impact of long-term oriented culture on corporate innovation, we further use substantive innovation (*Patent_Sub*) and strategic innovation (*Patent_Str*) to replace the variables of company innovation for testing. *Culture_LT* is the main independent variable, that is, the total words of long-term oriented culture of firms obtained from annual reports, then add 1, then take the natural logarithm.

Referring to Chemmanur et al. [43], among others, we used a set of control variables, including the age of the firm (*Lage*), the return on assets (*Roa*), the percentage of the top ten shareholders(*Top10*), the cash flow from operations (*Ocf*), the sales revenue growth ratio (*Growth*), the percentage of independent directors (*Indep*), the asset-liability ratio (*Lev*), the size of the firm (*Size*), the size of the board (*Board*), the combination of chairman and CEO (*Dual*), the nature of the property (*Soe*), and the year and industry fixed effects. We used the China Securities Regulatory Commission (CSRC) industry classification, with a two-digit code for the manufacturing sector and a one-digit code for other sectors. The Table 2 presents the definitions of the variables. Because there may be a lag in the role of long-term oriented culture on corporate innovation, and to further attenuate the effect of endogenous issues, all independent variables were lagged by one period in the tests used in this study.

#### 3.3.2. Hypothesis 2: The paths analysis of the long-term oriented culture and corporate innovation

**Fig 1:**
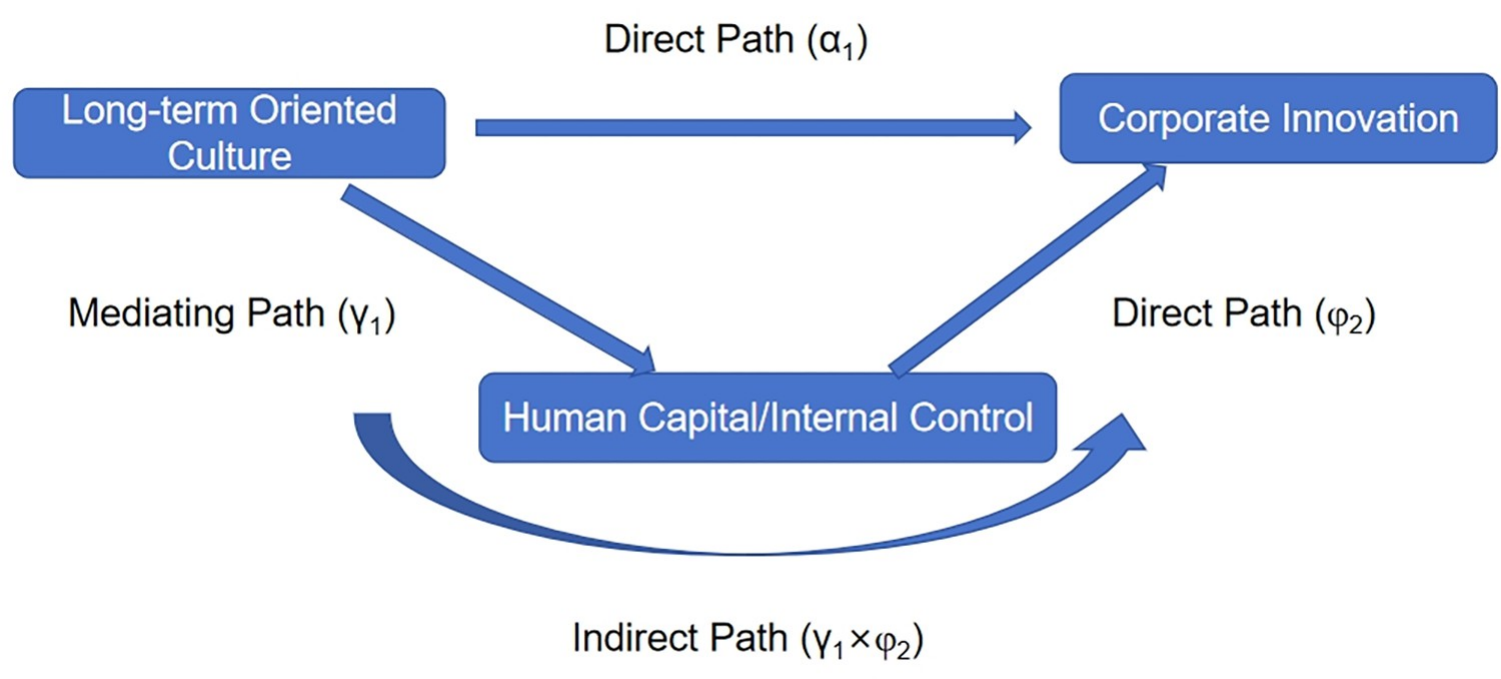
Path analysis.

According to hypothesis 2, we argue that long-term oriented culture promotes corporate innovation by enhancing the quality of human resources and internal control. That is, the quality of human resources and internal control are two specific paths that affect the relationship between long-term oriented culture and corporate innovation. Hilary et al. [44] pointed out that the effect of a mediating model can serve as a means of path analysis. The path analysis uses a structural equation model to answer how a source variable affects an outcome variable via their direct paths and indirect paths through mediating variables [45]. To examine the path role played by human resources quality and internal control between long-term oriented culture and corporate innovation, we also used a mediating effect approach (*X→Y*, *X→M*, *M→Y; X*:*long-term oriented culture*, *Y*:*corporate innovation*, *M*:*quality of human resources/internal control*) for path analysis (Fig 1).

The team human resources refers to all knowledge, skills and abilities of members in a team, among which education is an important dimension to measure team human resources. In view of this, we measure corporate human resources by the proportion of master’s degrees of corporate employees. In addition, the level of internal control of the firm was measured by the internal control indicator provided by the Dibble database.

In Eq (1), we examined the impact of long-term oriented culture on corporate innovation, which is the first step in mediating effect (*X→Y*). Next, we use Eq (2) to examine the effect of long-term oriented culture on human resources and internal control (*X→M*):

$${MasterRatio}_{i,t}({IC}_{i,t})=\gamma_{0}+\gamma_{1}{Culture\_LT}_{i,t}+\gamma {Controls}_{i,t}+\varepsilon_{i,t}$$ (2)

where *MasterRatio* is the proportion of master’s degrees of firm’s employees; *IC* is the internal control indicator of firms provided by the Dibble database.

Finally, we used the Eq (3) to examine the effect of human resources and internal control on corporate innovation (*M→Y)*:

$${Innovation}_{i,t+1}=\varphi_{0}+\varphi_{1}{Culture\_LT}_{i,t}+\varphi_{2}{MasterRatio}_{i,t}({IC}_{i,t})+\varphi {Controls}_{i,t}+\varepsilon_{i,t}$$ (3)

Baron and Kenny [45] proposed a whole set of testing procedures for mediating effect, which mainly can be used to investigate the coefficient values and significance of the developed models. First, γ_1_ and *φ*_*2*_ in model (2) and model (3) were tested gradually. If both are significant, it means that at least part of the effect *Culture_LT* on *Innovation* is through mediating variables (*MasterRatio* or *IC*). Hayes [46] divided the effect of a mediating model into direct and indirect effects, where φ_1_ reflects direct effects, and the *γ*_1_×*φ*_2_ reflects indirect effects.

#### 3.3.3. Hypothesis 3: The long term oriented culture, performance pressure, and corporate innovation

Hypothesis 3 of this study proposed that firms with a long-term oriented culture will still persist in innovation activities when facing performance pressure. To this end, we constructed Eq (4) for verification:

$${Innovation}_{i,t+1}=\beta_{0}+\beta_{1}{Culture\_LT}_{i,t}+\beta_{2}{Pressure}_{i,t}+\beta_{2}{{Culture\_LT}_{i,t}\times Pressure}_{i,t}{+\beta Controls}_{i,t}+\varepsilon_{i,t}$$ (4)

where *Performance* is the performance pressure faced by firms. We considered the performance pressures faced by the firm from both internal and external perspectives. From an internal perspective, *Pressure* is *P_Roa*, which is an indicator variable equal to 1 if the firm’s ROA is less than the average value in the industry where the firm is located, and otherwise is 0; From the external environment of the firm, performance pressure is represented by the product market competition faced by the firm and the degree of analyst tracking [47], that is, *Pressure* is *P_HHI* or *P_Analyst*. *P_HHI* is an indicator variable equal to 1 if the firm’s Herfindahl index is less than the average value in the industry where the firm is located, which means the firm’s product market competition is more intense, and otherwise is 0. *P_Analyst* is the natural logarithm of the total number of analysts tracked and add 1. For the test of Hypothesis 3, we mainly investigated whether the coefficient β_2_ of *Culture_LT×Performance* in the Eq (4) is significant.

### 3.4. Descriptive statistics and univariate analysis

We report the descriptive statistics and univariate analysis of the variables in Table 3. As shown in Table 3, the mean value of innovation capability (*Patent*) is 2.69, the minimum value is 0, and the maximum value is 6.75, indicating significant differences in innovation capability among different firms; the mean value of substantive innovation (*Patent_Sub*) is smaller than strategic innovation (*Patent_Str*), indicating that firms place more emphasis on strategic innovation.

**Table 3:** Descriptive statistics of the main variables.

Variable	N	Min	Median	Mean	Std	Max
*Patent*	15324	0.000	2.773	2.692	1.623	6.750
*Patent_Sub*	15324	0.000	1.792	1.854	1.456	5.855
*Patent_Str*	15324	0.000	2.197	2.147	1.605	6.174
*Culture_LT*	15324	4.419	5.617	5.611	0.455	6.815
*MasterRatio*	15324	0.000	0.010	0.028	0.050	0.539
*IC*	15324	0.000	6.726	6.472	1.299	8.637
*P_Roa*	15324	0.000	0.000	0.456	0.498	1.000
*P_HHI*	15324	0.000	1.000	0.673	0.469	1.000
*P_Analyst*	15324	0.000	1.609	1.597	1.129	3.434
*Lage*	15324	0.693	2.079	2.047	0.768	3.219
*Roa*	15324	-0.184	0.038	0.041	0.054	0.194
*Top10*	15324	0.234	0.599	0.588	0.148	0.903
*Ocf*	15324	-0.136	0.043	0.046	0.066	0.231
*Growth*	15324	-0.456	0.124	0.190	0.391	2.482
*Indep*	15324	0.250	0.375	0.386	0.076	0.600
*Lev*	15324	0.050	0.404	0.414	0.202	0.882
*Size*	15324	19.887	21.894	22.079	1.251	26.040
*Board*	15324	1.609	2.398	2.404	0.230	3.584
*Dual*	15324	0.000	0.000	0.294	0.456	1.000
*Soe*	15324	0.000	0.000	0.375	0.484	1.000

Table 4 provides univariate analysis of the core variables. We divided the sample into two groups based on the median of long-term oriented culture, one group with stronger long-term oriented culture and the other group with weaker long-term oriented culture. Table 4 shows that the innovation capability (*Patent*), substantive innovation (*Patent_Sub*), and strategic innovation (*Patent_Str*) of the group with stronger long-term oriented culture are significantly higher than those of the weaker group. This provides preliminary data support for the core hypothesis of this study.

**Table 4:** Univariate comparison.

Variable	Stronger long-term oriented culture group	Mean	Weaker long-term oriented culture group	Mean	Difference in mean
*Patent*	7757	2.851	7567	2.530	0.321***
*Patent_Sub*	7757	2.021	7567	1.684	0.337***
*Patent_Str*	7757	2.276	7567	2.016	0.260***

We reported the industry distribution of the sample and the descriptive statistics by industry in Table 5. This shows that the industries that disclose more long-term oriented cultural words are E, G, and R. At the same time, industries N, C4, and E that disclose more long-term oriented cultural words also have a higher number of patents. From a preliminary data analysis, there is a certain relationship between long-term oriented culture and corporate innovation.

**Table 5:** Descriptive statistics by industry.

Industry Code	Industry Name	N	Mean of *Culture_LT*	Mean of *Patent*
A	Agriculture, forestry, husbandry, and fishery	201	5.601	1.342
B	Mining	389	5.653	2.327
C1	Food processing and manufacturing	1080	5.556	2.255
C2	Petroleum, chemical, plastics, and rubber products	3250	5.574	2.361
C3	Machinery, equipment, and instrument manufacturing	6570	5.604	3.270
C4	Other manufacturing	223	5.685	3.158
D	Production and supply of electricity, steam, and tap water	322	5.552	2.085
E	Real estate	450	5.761	3.340
F	Wholesale and retail	420	5.613	1.757
G	Transportation and warehousing	230	5.746	1.873
H	Food and beverage	15	5.681	0.119
I	Information technology	1223	5.680	2.102
K	Non-metallic mineral products	163	5.538	1.585
L	Leasing and business service	155	5.670	1.418
M	Scientific and technical services	137	5.707	2.123
N	Water conservancy and environmental public facilities	178	5.647	2.843
P	Education	31	5.417	1.957
Q	Health and social work	50	5.614	1.110
R	Radio, film and television	150	5.716	1.574
S	Generals	87	5.643	2.733

## 4. Empirical results

### 4.1. The long-term oriented culture and corporate innovation

#### 4.1.1. Baseline regression results

We report the multivariable regression results of Eq (1) in Table 6. Firstly, columns (1) and (2) show that when the dependent variable is innovation capability (*Patent*), the coefficient of the long-term oriented culture (*Culture_LT*) is significant at the 1% level, indicating that the long-term oriented culture of firms significantly improves innovation ability. Hypothesis 1 is confirmed.

**Table 6:** Long-term oriented culture and corporate innovation.

	Innovation		Substantive Innovation		Strategic Innovation	
	(1)	(2)	(3)	(4)	(5)	(6)
	*Patent*	*Patent*	*Patent_Sub*	*Patent_Sub*	*Patent_Str*	*Patent_Str*
***Culture_LT***	0.302***	0.274***	0.454***	0.272***	0.089***	0.197***
	(10.475)	(5.289)	(17.578)	(5.775)	(3.099)	(3.974)
*Lage*		-0.089**		-0.092**		-0.073**
		(-2.304)		(-2.562)		(-2.005)
*Roa*		3.671***		3.011***		3.085***
		(10.421)		(9.056)		(8.933)
*Top10*		-0.512***		-0.637***		-0.214
		(-2.888)		(-3.844)		(-1.256)
*Ocf*		-0.374		-0.322		-0.290
		(-1.427)		(-1.351)		(-1.158)
*Growth*		0.042		0.056*		0.024
		(1.323)		(1.908)		(0.790)
*Indep*		0.375*		0.240		0.370*
		(1.705)		(1.143)		(1.766)
*Lev*		0.037		-0.131		0.255**
		(0.280)		(-1.046)		(2.012)
*Size*		0.542***		0.521***		0.466***
		(21.038)		(20.802)		(18.603)
*Board*		0.046		0.063		-0.004
		(0.561)		(0.822)		(-0.055)
*Dual*		0.029		0.036		0.005
		(0.683)		(0.884)		(0.126)
*Soe*		0.075		0.149**		0.004
		(1.233)		(2.538)		(0.075)
*_cons*	0.986***	-12.254***	-0.636***	-12.160***	1.575***	-10.734***
	(6.071)	(-20.949)	(-4.372)	(-21.194)	(9.760)	(-19.733)
*Industry*	No	Yes	No	Yes	No	Yes
*Year*	No	Yes	No	Yes	No	Yes
N	15324	15324	15324	15324	15324	15324
adj. R^2^	0.007	0.354	0.020	0.315	0.001	0.367

Notes

***, **, and * indicate statistical significance at the 1%, 5%, and 10% level, respectively, using two-tailed tests and standard errors adjusted for firm-clustering.

Next, we differentiated innovation into substantive innovation and strategic innovation, and examined the differences in the impact of long-term oriented culture on these two types of innovation behaviors. Columns (3) to (6) in Table 6 show that when the dependent variables are substantive innovation (*Patent_Sub*) and strategic innovation *(Patent_Str*), the coefficient of the long-term oriented culture (*Culture_LT*) is significant at the 1% level. However, without considering control variables, the coefficient of *Culture_LT* in column (3) is 0.454 and the coefficient of *Culture_LT* in column (5) is 0.089, indicating that firms with a long-term oriented culture pay more attention to inventive innovation inputs and outputs that have substantive improvements for the firms, which is more beneficial to the long-term development of the company. After controlling for other factors, there is still a significant difference. As shown in columns (4) and (6), the coefficient value of *Culture_LT* in column (4) is 0.272 and the coefficient value of *Culture_LT* in column (6) is 0.197, indicating that the coefficient value of *Culture_LT* in column (5) is 38.07% higher than in column (6). This shows that after considering other factors, firms with a long-term oriented culture may engage in both substantive and strategic innovation activities, but they will pay more attention to substantive innovation.

#### 4.1.2. Robustness tests

*(1) The sustained impact of long-term oriented culture*. Corporate innovation requires continuous investment, and it takes a long time to convert innovation investment into innovation output. We used the innovation output of the t+1 period in the main regression test, which means lagging all independent variables by one period. To further investigate the sustained impact of long-term oriented culture on corporate innovation, we used innovation data from periods t+2 and t+3 to test. Columns (1) to (6) of Table 7 show that the coefficients of long-term oriented culture (*Culture_LT*) are still significantly positive, indicating that long-term oriented culture promotes innovation and the robustness of the results.

**Table 7:** Robustness tests:The sustained impact of long-term oriented culture.

	t+2 period			t+3 period		
	(1)	(2)	(3)	(4)	(5)	(6)
	*Patent*_*t+2*_	*Patent_Sub*_*t+2*_	*Patent_Str*_*t+2*_	*Patent*_*t+3*_	*Patent_Sub*_*t+3*_	*Patent_Str*_*t+3*_
***Culture_LT***	0.352***	0.369***	0.250***	0.333***	0.360***	0.228***
	(5.426)	(6.155)	(4.041)	(5.019)	(5.813)	(3.647)
*Lage*	-0.105**	-0.102**	-0.091**	-0.117**	-0.115***	-0.099**
	(-2.421)	(-2.536)	(-2.235)	(-2.525)	(-2.674)	(-2.250)
*Roa*	4.176***	3.324***	3.745***	4.265***	3.380***	3.823***
	(9.585)	(8.100)	(8.852)	(9.470)	(7.898)	(8.732)
*Top10*	-0.487**	-0.595***	-0.224	-0.463**	-0.576***	-0.205
	(-2.546)	(-3.319)	(-1.228)	(-2.327)	(-3.062)	(-1.078)
*Ocf*	-0.221	-0.216	-0.153	0.023	0.143	-0.011
	(-0.761)	(-0.806)	(-0.552)	(0.075)	(0.506)	(-0.036)
*Growth*	0.060*	0.097***	0.025	0.095**	0.110***	0.079**
	(1.710)	(2.983)	(0.760)	(2.569)	(3.137)	(2.296)
*Indep*	0.345	0.230	0.358	0.408	0.284	0.492**
	(1.418)	(0.986)	(1.535)	(1.580)	(1.137)	(2.005)
*Lev*	0.022	-0.134	0.257*	0.005	-0.155	0.252*
	(0.148)	(-0.977)	(1.846)	(0.036)	(-1.077)	(1.753)
*Size*	0.521***	0.504***	0.447***	0.502***	0.485***	0.427***
	(19.043)	(18.781)	(16.868)	(17.605)	(17.197)	(15.601)
*Board*	0.047	0.062	0.015	0.058	0.095	0.021
	(0.515)	(0.727)	(0.166)	(0.607)	(1.036)	(0.232)
*Dual*	0.010	0.046	-0.031	0.000	0.041	-0.049
	(0.220)	(1.002)	(-0.683)	(0.004)	(0.841)	(-1.018)
*Soe*	0.096	0.179***	0.015	0.095	0.182***	0.008
	(1.473)	(2.818)	(0.238)	(1.386)	(2.731)	(0.118)
*_cons*	-11.983***	-12.134***	-10.480***	-11.306***	-11.577***	-9.825***
	(-18.572)	(-19.118)	(-17.738)	(-16.899)	(-17.443)	(-16.204)
*Industry*	Yes	Yes	Yes	Yes	Yes	Yes
*Year*	Yes	Yes	Yes	Yes	Yes	Yes
*N*	12802	12802	12802	11024	11024	11024
*adj*. *R*^*2*^	0.343	0.305	0.358	0.339	0.296	0.361

Notes

***, **, and * indicate statistical significance at the 1%, 5%, and 10% level, respectively, using two-tailed tests and standard errors adjusted for firm-clustering.

*(2) Replacing the main variables*.We first replaced the measurement of long-term oriented culture. The long-term oriented culture in this study was obtained through the text processing of the annual report. To eliminate deviations in the processing of text information, this article divided the number of words with long-term oriented culture into five level (*Culture_LT_5*) by each industry each year. Specifically, *Culture_LT_5* divides *Culture_LT* into 1, 2, 3, 4 and 5 levels according to the long-term oriented culture level of the industry in which the firm is located every year. The regression results are shown in columns (1) to (3) of Table 8. In the regression results shown without and with consideration of the control variables, the regression coefficients of long-term oriented culture (*Culture_LT_5*) are significant at the 1% level, indicating the robustness of the results. Second, the measurement of corporate innovation was replaced. As mentioned earlier, R&D expenditure is still an important indicator of corporate innovation input. Therefore, from the perspective of company innovation input, we took R&D expenditure as the dependent variable to retest the impact of long-term oriented culture on corporate innovation. The results in column (4) of Table 8 indicate that a long-term oriented culture can also affect corporate innovation input, indicating the robustness of the conclusion.

**Table 8:** Robustness tests: Replacing the measurement of main variables and considering the R&D.

	Replacing the long-term oriented culture			Replacing the corporate innovation	Controlling the R&D		
	(1)	(2)	(3)	(4)	(5)	(6)	(7)
	*Patent*	*Patent_Sub*	*Patent_Str*	*RD*	*Patent*	*Patent_Sub*	*Patent_Str*
***Culture_LT_5***	0.070***	0.077***	0.051***				
	(5.678)	(6.766)	(4.246)				
***Culture_LT***				0.002***	0.228***	0.220***	0.169***
				(3.237)	(4.527)	(4.948)	(3.428)
*RD*					21.853***	24.538***	13.500***
					(15.893)	(19.074)	(9.586)
*Lage*	-0.083**	-0.083**	-0.069*	-0.002***	-0.049	-0.046	-0.048
	(-2.136)	(-2.317)	(-1.875)	(-3.948)	(-1.338)	(-1.393)	(-1.358)
*Roa*	3.656***	2.998***	3.075***	0.052***	2.537***	1.738***	2.385***
	(10.387)	(9.039)	(8.903)	(10.369)	(7.552)	(5.681)	(7.076)
*Top10*	-0.508***	-0.633***	-0.211	-0.004**	-0.417**	-0.530***	-0.155
	(-2.869)	(-3.825)	(-1.242)	(-2.236)	(-2.499)	(-3.455)	(-0.939)
*Ocf*	-0.356	-0.298	-0.277	0.018***	-0.760***	-0.756***	-0.529**
	(-1.358)	(-1.251)	(-1.104)	(5.590)	(-3.059)	(-3.384)	(-2.170)
*Growth*	0.038	0.050*	0.020	-0.000	0.051*	0.066**	0.029
	(1.187)	(1.714)	(0.684)	(-1.219)	(1.701)	(2.407)	(1.011)
*Indep*	0.370*	0.234	0.367*	0.005*	0.272	0.126	0.307
	(1.685)	(1.116)	(1.749)	(1.955)	(1.305)	(0.642)	(1.497)
*Lev*	0.044	-0.123	0.260**	-0.001	0.064	-0.100	0.271**
	(0.329)	(-0.988)	(2.051)	(-0.819)	(0.512)	(-0.872)	(2.201)
*Size*	0.537***	0.513***	0.462***	-0.002***	0.581***	0.564***	0.490***
	(20.765)	(20.452)	(18.401)	(-6.193)	(24.319)	(24.618)	(20.340)
*Board*	0.046	0.063	-0.004	0.000	0.043	0.060	-0.006
	(0.559)	(0.819)	(-0.057)	(0.124)	(0.567)	(0.867)	(-0.080)
*Dual*	0.025	0.032	0.003	0.001	0.014	0.019	-0.004
	(0.608)	(0.786)	(0.070)	(1.264)	(0.352)	(0.517)	(-0.094)
*Soe*	0.076	0.151**	0.005	0.000	0.066	0.139***	-0.001
	(1.244)	(2.566)	(0.083)	(0.557)	(1.170)	(2.633)	(-0.027)
*_cons*	-11.029***	-10.914***	-9.850***	0.031***	-12.925***	-12.913***	-11.148***
	(-20.237)	(-20.334)	(-19.235)	(4.225)	(-23.989)	(-24.934)	(-21.342)
*Industry*	Yes	Yes	Yes	Yes	Yes	Yes	Yes
*Year*	Yes	Yes	Yes	Yes	Yes	Yes	Yes
*N*	15324	15324	15324	15324	15324	15324	15324
*adj*. *R*^*2*^	0.355	0.317	0.368	0.292	0.394	0.377	0.382

Notes

***, **, and * indicate statistical significance at the 1%, 5%, and 10% level, respectively, using two-tailed tests and standard errors adjusted for firm-clustering.

*(3) Controlling for the effect of R&D investment*. As mentioned earlier, R&D expenditure is an important indicator of corporate innovation input, and therefore, it is an important factor affecting innovation output. Thus, we added the control variable R&D expenditure (*RD*) in Eq (1) and repeated the regression. The results are shown in columns (5) to (7) of Table 8, and the results are still robust.

*(4) Instrumental variables*. The endogeneity of reverse causality can be addressed to some extent by the instrumental variables approach. That is, with a high innovation capability in a firm may lead to a higher long-term oriented culture. We the mean value of long-term oriented culture of other firms in the same region and industry as the instrumental variable (*IV*) of long-term oriented culture of firms. The results are shown in columns (1) to (4) of Table 9. Columns (1) to (4) show the regression results of two stages of the instrumental variable of long-term oriented culture. Column (1) is the regression result of the first stage, and it is found that the instrumental variable of long-term oriented culture is significantly and positively correlated with long-term oriented culture and its coefficient is significant at the 1% level with an F-value of 501.63, which is much greater than 10, and the instrumental variable is effectively selected. Columns (2) to (4) are the results of the second stage regression, and the coefficient of *Culture_LT* is still significantly positive at the 1% level, indicating that the results are robust.

**Table 9:** Robustness tests: Endogeneity problems.

	Instrumental variable				PSM		
	(1)	(2)	(3)	(4)	(5)	(6)	(7)
	*Culture_LT*	*Patent*	*Patent_Sub*	*Patent_Str*	*Patent*	*Patent_Sub*	*Patent_Str*
***Culture_LT IV***	0.431***						
	(19.945)						
***Culture_LT***		1.147***	1.232***	0.852***			
		(5.100)	(5.860)	(3.895)			
***DumCulture_LT***					0.130***	0.155***	0.082**
					(3.989)	(5.127)	(2.575)
*Lage*	-0.049***	-0.036	-0.034	-0.034	-0.052	-0.057	-0.041
	(-10.526)	(-1.464)	(-1.452)	(-1.403)	(-1.258)	(-1.485)	(-1.038)
*Roa*	-0.128**	3.776***	3.126***	3.164***	3.813***	3.182***	3.270***
	(-2.254)	(14.709)	(13.026)	(12.676)	(10.020)	(8.906)	(8.721)
*Top10*	-0.003	-0.510***	-0.635***	-0.213**	-0.482***	-0.595***	-0.195
	(-0.127)	(-5.777)	(-7.690)	(-2.478)	(-2.623)	(-3.485)	(-1.097)
*Ocf*	-0.131***	-0.252	-0.188	-0.199	-0.458	-0.487*	-0.334
	(-3.146)	(-1.330)	(-1.062)	(-1.079)	(-1.629)	(-1.927)	(-1.240)
*Growth*	0.031***	0.012	0.023	0.001	0.056	0.081**	0.031
	(4.788)	(0.388)	(0.798)	(0.033)	(1.586)	(2.531)	(0.931)
*Indep*	0.017	0.344**	0.206	0.347**	0.238	0.193	0.143
	(0.506)	(2.335)	(1.499)	(2.426)	(1.049)	(0.888)	(0.664)
*Lev*	-0.017	0.056	-0.110	0.269***	0.108	-0.084	0.351***
	(-1.005)	(0.746)	(-1.581)	(3.707)	(0.774)	(-0.639)	(2.601)
*Size*	0.073***	0.473***	0.445***	0.414***	0.506***	0.482***	0.425***
	(26.539)	(21.923)	(22.060)	(19.735)	(17.138)	(17.245)	(14.990)
*Board*	0.011	0.042	0.059	-0.007	0.091	0.111	0.034
	(0.948)	(0.829)	(1.245)	(-0.148)	(1.078)	(1.391)	(0.427)
*Dual*	0.017***	0.010	0.016	-0.008	0.031	0.049	-0.006
	(3.055)	(0.410)	(0.663)	(-0.341)	(0.711)	(1.204)	(-0.148)
*Soe*	-0.027***	0.098***	0.175***	0.021	0.062	0.156**	-0.021
	(-4.345)	(3.386)	(6.439)	(0.761)	(0.984)	(2.534)	(-0.344)
*_cons*	1.154***	-15.289***	-15.497***	-13.009***	-10.335***	-10.248***	-9.029***
	(8.767)	(-18.447)	(-20.001)	(-16.144)	(-16.983)	(-17.616)	(-15.902)
*Industry*	Yes	Yes	Yes	Yes	Yes	Yes	Yes
*Year*	Yes	Yes	Yes	Yes	Yes	Yes	Yes
*N*	15324	15324	15324	15324	12528	12528	12528
*adj*. *R*^*2*^	0.572	0.328	0.276	0.352	0.314	0.269	0.335

Notes

***, **, and * indicate statistical significance at the 1%, 5%, and 10% level, respectively, using two-tailed tests and standard errors adjusted for firm-clustering.

*(5) Propensity score matching*. We used propensity score matching (PSM) samples to examine Eq (1) to mitigate potential endogeneity issues. First, we calculated the mean of long-term oriented culture based on the each year and each industry in the sample. If the firm was greater than the mean, it was included in the treatment firm group; if it was less than the mean, it was included with the control firms. Secondly, we constructed a logit model using *Lage*, *Roa*, *Top10*, *Ocf*, *Growth*, *Indep*, *Lev*, *Size*, *Board*, *Dual* and *Soe* as explanatory variables. This approach ensures that the characteristics that affect innovation do not significantly differ between the treatment firms and control firms. Based on the propensity scores calculated, we then conducted a 1:1 matching of treatment and control using the nearest-neighbor and non-replacement methods. We had 6,264 samples for the treatment group, and 6,264 samples for the control group. Finally, we repeated the regression using PSM samples, and the results are shown in columns (5)-(7) of Table 9. The coefficient of long-term oriented culture (*DumCulture_LT*) is still significantly positive, indicating the robustness of our results.

### 4.2. Long-term oriented culture and corporate innovation: Paths analysis

The previous empirical results indicate that the long-term oriented culture of firms is beneficial for improving their innovation capabilities. The more important question is: What are the paths by which the long-term oriented culture of firms to enhances their innovation capabilities? Therefore, we proposed hypothesis 2, which suggests that the long-term oriented culture promotes innovation by enhancing human resources and improving the quality of internal control within the firms. In accordance with Hayes [46], we used a mediating effect approach for testing.

#### 4.2.1 The path analysis of human resources

We tested whether human resources plays a mediating effect in the relationship between long-term oriented culture and corporate innovation, as shown in Table 10. Column (1) of Table 10 is the regression result of Eq (2), which shows that the coefficient of long-term oriented culture (*Culture_LT*) is 0.010 and significant at the 1% level, indicating that the firms with a long-term oriented culture can improve human resources, that is, the proportion of employees with higher education. Columns (2) to (4) are the regression results of Eq (3), where the explanatory variables are innovation (*Patent*), substantive innovation (*Patent_Sub*), and strategic innovation (*Patent_Str*), respectively, where the coefficient of *Culture_LT* in column (2) is 0.248 and the coefficient of human resources is 2.679, both of which are significant at the 1% level. We classified mediation effects into direct effects and indirect effects based on Hayes [46]. According to the regression results in Table 10, the direct effect shown as φ_1_ is 0.248, and the indirect effect is γ_1_×φ_2_ = 0.01×2.679 = 0.0267. The coefficients of *Culture_LT* and human resources in column (3) are both significantly positive; however, in column (4), the coefficient of *Culture_LT* is significantly positive at the 1% level, but the coefficient of human resources is insignificant; further, the Sobel [48] test was conducted and the p-value was found to be greater than 0.01, indicating that the mediating effect does not hold in long-term oriented culture and strategic innovation. Thus, the firms with the long-term oriented culture promote the real level of innovation by increasing human resources but not strategic innovation.

**Table 10:** The path analysis of human resources.

	Step1	Step2		
	(1)	(2)	(3)	(4)
	*MasterRatio*	*Patent*	*Patent_Sub*	*Patent_Str*
***Culture_LT***	0.010***	0.248***	0.230***	0.196***
	(5.54)	(4.84)	(4.99)	(3.97)
***MasterRatio***		2.679***	4.444***	0.090
		(6.78)	(10.21)	(0.23)
*Lage*	-0.004**	-0.079**	-0.076**	-0.073**
	(-2.50)	(-2.06)	(-2.14)	(-1.99)
*Roa*	0.079***	3.459***	2.658***	3.078***
	(5.55)	(9.97)	(8.32)	(8.97)
*Top10*	-0.023***	-0.449**	-0.533***	-0.212
	(-3.58)	(-2.55)	(-3.29)	(-1.24)
*Ocf*	-0.043***	-0.259	-0.131	-0.286
	(-5.01)	(-1.00)	(-0.56)	(-1.14)
*Growth*	0.003**	0.034	0.043	0.023
	(2.48)	(1.09)	(1.49)	(0.78)
*Indep*	-0.001	0.376*	0.243	0.370*
	(-0.08)	(1.72)	(1.18)	(1.77)
*Lev*	-0.025***	0.104	-0.020	0.257**
	(-4.20)	(0.78)	(-0.17)	(2.02)
*Size*	0.002**	0.536***	0.511***	0.465***
	(2.02)	(20.99)	(20.73)	(18.57)
*Board*	0.005	0.033	0.042	-0.005
	(1.60)	(0.41)	(0.56)	(-0.06)
*Dual*	0.001	0.027	0.033	0.005
	(0.44)	(0.65)	(0.83)	(0.12)
*Soe*	0.018***	0.028	0.070	0.003
	(6.96)	(0.46)	(1.23)	(0.05)
*_cons*	-0.084***	-12.028***	-11.785***	-10.726***
	(-3.73)	(-20.94)	(-21.21)	(-19.73)
*Industry*	Yes	Yes	Yes	Yes
*Year*	Yes	Yes	Yes	Yes
*N*	15324	15324	15324	15324
*adj*. *R*^*2*^	0.153	0.360	0.334	0.367

Notes

***, **, and * indicate statistical significance at the 1%, 5%, and 10% level, respectively, using two-tailed tests and standard errors adjusted for firm-clustering.

#### 4.2.2. The path analysis of internal control quality

We tested whether internal control plays a mediating effect in the relationship between long-term oriented culture and innovation. The results are shown in Table 11, where column (1) is the regression result of Eq (2), which shows that the coefficient of long-term oriented culture (*Culture_LT*) is 0.102 and significant at the 1% level, indicating that long-term oriented culture enhances the quality of internal control; columns (2), (3), and (4) are the regression results of Eq (3), and the explanatory variables are innovation (*Patent*), substantive innovation (*Patent_Sub*), and strategic innovation (*Patent_Str*), respectively. The coefficient of long-term oriented culture in column (2) is 0.267 and the coefficient of internal control quality is 0.068, and both are significant at the 1% level. The direct effect shown is as φ_1_ is 0.267 and the indirect effect is γ_1_×φ_2_ = 0.102×0.068 = 0.007. The coefficients of long-term oriented culture (*Culture_LT)* and internal control quality (*IC*) in columns (3) and (4) are still positive and significant, which indicates that long-term oriented culture further promotes innovation by improving the quality of internal control.

**Table 11:** The path analysis of internal control.

	Step1	Step2		
	(1)	(2)	(3)	(4)
	*IC*	*Patent*	*Patent_Sub*	*Patent_Str*
***Culture_LT***	0.102***	0.267***	0.266***	0.191***
	(2.72)	(5.17)	(5.67)	(3.87)
***IC***		0.068***	0.059***	0.057***
		(5.39)	(5.12)	(4.67)
*Lage*	-0.204***	-0.075*	-0.080**	-0.062*
	(-7.26)	(-1.94)	(-2.22)	(-1.68)
*Roa*	7.756***	3.140***	2.552***	2.640***
	(19.42)	(8.77)	(7.66)	(7.51)
*Top10*	-0.136	-0.502***	-0.628***	-0.206
	(-1.25)	(-2.84)	(-3.80)	(-1.21)
*Ocf*	-0.690***	-0.327	-0.281	-0.251
	(-2.93)	(-1.25)	(-1.18)	(-1.00)
*Growth*	0.110***	0.035	0.049*	0.017
	(2.84)	(1.09)	(1.69)	(0.58)
*Indep*	0.617***	0.333	0.204	0.335
	(4.36)	(1.52)	(0.97)	(1.60)
*Lev*	-0.544***	0.074	-0.098	0.286**
	(-4.32)	(0.56)	(-0.79)	(2.26)
*Size*	0.258***	0.524***	0.505***	0.451***
	(12.32)	(20.22)	(20.06)	(17.84)
*Board*	-0.208***	0.060	0.075	0.008
	(-3.23)	(0.74)	(0.98)	(0.10)
*Dual*	-0.036	0.031	0.038	0.007
	(-1.33)	(0.74)	(0.94)	(0.18)
*Soe*	0.067*	0.071	0.145**	0.000
	(1.69)	(1.16)	(2.48)	(0.01)
*_cons*	1.079**	-12.328***	-12.224***	-10.796***
	(2.36)	(-21.23)	(-21.44)	(-19.95)
*Industry*	Yes	Yes	Yes	Yes
*Year*	Yes	Yes	Yes	Yes
*N*	15324	15324	15324	15324
*adj*. *R*^*2*^	0.196	0.357	0.317	0.369

Notes

***, **, and * indicate statistical significance at the 1%, 5%, and 10% level, respectively, using two-tailed tests and standard errors adjusted for firm-clustering.

### 4.3. Long-term oriented culture, performance pressure and corporate innovation

We further tested hypothesis 3 for whether companies with a long-term oriented culture will persist in innovation when facing performance pressure. We tested from three perspectives of performance pressures. The first perspective was the profit pressure faced by the firms; the second perspective was the external product market competition faced by the firms; and the third perspective was the pressure on the firms to receive analyst attention.

#### 4.3.1. Performance pressure from the perspective of corporate profitability

Firstly, we tested the performance pressure affecting on the relationship between long-term oriented culture and corporate innovation from the perspective of corporate profitability. The regression results of Eq (4) are shown in Table 12. The performance pressure of corporate profitability faced by firms is represented by *P_Roa*. *P_Roa* is an indicator variable equal to 1 if the firm’s ROA is less than the average value in the industry where the firm is located, and otherwise is 0. We mainly focused on the coefficient of the *Culture_LT×P_Roa*. As shown in columns (1) to (3) of Table 12, when the dependent variable is *Patent*, *Patent_Sub*, or *Patent_Str*, the coefficient of the *Culture_LT×P_Roa* is significantly positive, indicating that the long-term oriented culture makes firms more persistent in innovation under profitability performance pressure, which supports hypothesis 3.

**Table 12:** Performance pressure from the perspective of corporate profitability.

	(1)	(2)	(3)
	*Patent*	*Patent_Sub*	*Patent_Str*
*Culture_LT*	0.205***	0.225***	0.147***
	(3.667)	(4.424)	(2.707)
*P_Roa*	-0.954***	-0.685**	-0.685**
	(-2.956)	(-2.360)	(-2.168)
***Culture_LT×P_Roa***	0.144**	0.095*	0.105*
	(2.525)	(1.849)	(1.877)
*Lage*	-0.087**	-0.089**	-0.072**
	(-2.260)	(-2.504)	(-1.973)
*Roa*	2.892***	2.204***	2.570***
	(7.727)	(6.406)	(6.924)
*Top10*	-0.531***	-0.654***	-0.227
	(-2.993)	(-3.948)	(-1.331)
*Ocf*	-0.424	-0.368	-0.324
	(-1.623)	(-1.548)	(-1.297)
*Growth*	0.036	0.051*	0.020
	(1.144)	(1.727)	(0.661)
*Indep*	0.379*	0.242	0.373*
	(1.727)	(1.153)	(1.782)
*Lev*	0.071	-0.100	0.278**
	(0.534)	(-0.804)	(2.199)
*Size*	0.541***	0.520***	0.465***
	(21.066)	(20.845)	(18.606)
*Board*	0.044	0.062	-0.006
	(0.540)	(0.807)	(-0.073)
*Dual*	0.028	0.035	0.005
	(0.672)	(0.876)	(0.117)
*Soe*	0.082	0.155***	0.009
	(1.344)	(2.644)	(0.154)
*_cons*	-11.817***	-11.853***	-10.419***
	(-19.769)	(-20.332)	(-18.582)
*Industry*	Yes	Yes	Yes
*Year*	Yes	Yes	Yes
*N*	15324	15324	15324
*adj*. *R*^*2*^	0.356	0.317	0.368

Notes

***, **, and * indicate statistical significance at the 1%, 5%, and 10% level, respectively, using two-tailed tests and standard errors adjusted for firm-clustering.

#### 4.3.2. Performance pressure from the perspective of product market competition

Product market competition serves as an important external governance mechanism, yet when companies face tougher market competition, there is still pressure for performance to decline. At this point, is a long-term oriented culture more conducive to corporate adherence to innovation to enhance their competitiveness? We used product market competition as an external measure of performance pressure to test the effect on the relationship between long-term oriented culture and corporate innovation. The regression results based on Eq (4) are shown in Table 13. The performance pressure of product market competition faced by firms is represented by *P_HHI*. *P_HHI* is an indicator variable equal to 1 if the firm’s Herfindahl index is less than the average value in the industry where the firm is located, which means the firm’ product market competition is more intense, and otherwise is 0. We mainly focus on the coefficient of the *Culture_LT×P_HHI*. As shown in columns (1) to (3) of Table 13, when the dependent variable is *Patent*, *Patent_Sub*, or *Patent_Str*, the coefficient of the *Culture_LT×P_HHI* is significantly positive, indicating that long-term oriented culture makes firms more persistent in innovation under pressure from product market competition, which supports hypothesis 3.

**Table 13:** Performance pressure from the perspective of product market competition.

	(1)	(2)	(3)
	*Patent*	*Patent_Sub*	*Patent_Str*
*Culture_LT*	0.160**	0.195***	0.086
	(2.394)	(3.267)	(1.318)
*P_HHI*	-0.808**	-0.411	-0.929***
	(-2.207)	(-1.289)	(-2.610)
***Culture_LT×P_HHI***	0.163**	0.105*	0.166***
	(2.557)	(1.863)	(2.681)
*Lage*	-0.086**	-0.088**	-0.072**
	(-2.229)	(-2.463)	(-1.978)
*Roa*	3.667***	2.990***	3.098***
	(10.423)	(9.019)	(8.987)
*Top10*	-0.502***	-0.622***	-0.212
	(-2.846)	(-3.784)	(-1.251)
*Ocf*	-0.348	-0.284	-0.285
	(-1.330)	(-1.196)	(-1.137)
*Growth*	0.044	0.057**	0.025
	(1.384)	(1.966)	(0.844)
*Indep*	0.382*	0.244	0.378*
	(1.739)	(1.162)	(1.807)
*Lev*	0.047	-0.109	0.250**
	(0.353)	(-0.883)	(1.973)
*Size*	0.543***	0.523***	0.466***
	(21.194)	(21.014)	(18.660)
*Board*	0.049	0.069	-0.005
	(0.598)	(0.898)	(-0.063)
*Dual*	0.029	0.035	0.007
	(0.700)	(0.882)	(0.161)
*Soe*	0.069	0.139**	0.003
	(1.130)	(2.390)	(0.056)
*_cons*	-11.737***	-11.898***	-10.137***
	(-18.565)	(-19.736)	(-17.145)
*Industry*	Yes	Yes	Yes
*Year*	Yes	Yes	Yes
*N*	15324	15324	15324
*adj*. *R*^*2*^	0.356	0.318	0.368

Notes

***, **, and * indicate statistical significance at the 1%, 5%, and 10% level, respectively, using two-tailed tests and standard errors adjusted for firm-clustering.

#### 4.3.3. Performance pressure from the perspective of analyst attention

As an important intermediary force, analysts have to some extent alleviated the problem of information asymmetry inside and outside the firms. However, analyst attention and analysis of the firms, especially in predicting profits, can also lead to performance pressure for the firms to achieve analyst predictions [47]. Therefore, we used analyst attention as a proxy for firms facing external performance pressure. The regression results based on Eq (4) are shown in Table 14. The performance pressure of analyst attention faced by firms is represented by *P_Analyst*. *P_Analyst* is the natural logarithm of the total number of analysts tracked and add 1. We mainly focused on the coefficient of the *Culture_LT×P_Analyst*. As shown in columns (1) to (3) of Table 14, when the dependent variable is *Patent*, *Patent_Sub*, or *Patent_Str*, the coefficient of the *Culture_LT×P_Analyst* is significantly positive, indicating that the long-term oriented culture makes firms more persistent in innovation under pressure from analyst attention, which supports hypothesis 3.

**Table 14:** Performance pressure from the perspective of analyst attention.

	(1)	(2)	(3)
	*Patent*	*Patent_Sub*	*Patent_Str*
*Culture_LT*	0.261***	0.259***	0.190***
	(5.005)	(5.467)	(3.786)
*P_Analyst*	0.152***	0.160***	0.100***
	(7.594)	(8.553)	(5.065)
***Culture_LT×P_Analyst***	0.080***	0.085***	0.065**
	(2.866)	(3.518)	(2.405)
*Lage*	-0.064*	-0.065*	-0.057
	(-1.650)	(-1.817)	(-1.559)
*Roa*	2.620***	1.905***	2.394***
	(7.508)	(5.872)	(6.926)
*Top10*	-0.487***	-0.611***	-0.196
	(-2.773)	(-3.728)	(-1.158)
*Ocf*	-0.456*	-0.408*	-0.345
	(-1.739)	(-1.717)	(-1.375)
*Growth*	0.032	0.045	0.017
	(1.008)	(1.561)	(0.571)
*Indep*	0.380*	0.246	0.373*
	(1.740)	(1.178)	(1.786)
*Lev*	0.093	-0.072	0.290**
	(0.704)	(-0.583)	(2.296)
*Size*	0.465***	0.440***	0.415***
	(16.771)	(16.259)	(15.258)
*Board*	0.064	0.082	0.009
	(0.788)	(1.079)	(0.110)
*Dual*	0.025	0.032	0.003
	(0.599)	(0.795)	(0.068)
*Soe*	0.112*	0.188***	0.029
	(1.841)	(3.229)	(0.496)
*_cons*	-9.227***	-9.065***	-8.644***
	(-15.754)	(-15.773)	(-15.423)
*Industry*	Yes	Yes	Yes
*Year*	Yes	Yes	Yes
*N*	15324	15324	15324
*adj*. *R*^*2*^	0.362	0.326	0.371

Notes

***, **, and * indicate statistical significance at the 1%, 5%, and 10% level, respectively, using two-tailed tests and standard errors adjusted for firm-clustering.

## 5. Conclusions

We extracted the cultural element of long-term orientation from Chinese listed firm’s annual reports, then argued and testified about long-term orientation can help firms to hang onto risky decisions, especially on innovation, when firms are under performance pressure. We reached three main conclusions. First, for both substantive and strategic innovation, the higher the degree of long-term oriented culture a firm has, the stronger innovation capability the firm shows. Second, in considering specific paths, we find that long-term oriented culture can improve employee educational qualification to promote corporate innovation, especially substantive innovative capacity rather than strategic innovative capacity. At the same time, long-term oriented culture can improve the internal control system to promote both kinds of innovation. Third, an extended analysis shows that when firms are subjected to different kinds of performance pressure in their business processes, including internal performance pressure such as low profit, and external performance pressure, such as fierce market competition or high analyst tracking, firms with a higher long-term oriented culture will be more innovative with respect to both kinds of innovations.
